# Draft Genome Sequence of the Starmerella bacillaris (syn., Candida zemplinina) Type Strain CBS 9494

**DOI:** 10.1128/MRA.00872-18

**Published:** 2018-07-26

**Authors:** Alberto Luis Rosa, Cécile Miot-Sertier, Yec’han Laizet, Franck Salin, Matthias Sipiczki, Marina Bely, Isabelle Masneuf-Pomarede, Warren Albertin

**Affiliations:** a Laboratorio de Genética y Biología Molecular, IRNASUS-CONICET, Facultad de Ciencias Químicas, Universidad Católica de Córdoba, Córdoba, Argentina; b ISVV, Oenology Research Unit EA 4577, USC 1366 INRA, Bordeaux INP, University of Bordeaux, Villenave d’Ornon, France; c UMR Biodiversity of Genes and Ecosystems, Genomics Platform, INRA, Cestas, France; d Department of Genetics and Applied Microbiology, University of Debrecen, Debrecen, Hungary; e Bordeaux Sciences Agro, Gradignan, France; f ENSCBP, Bordeaux INP, Pessac, France; Queens College

## Abstract

Starmerella bacillaris is an ascomycetous yeast ubiquitously present in grapes and fermenting grape musts. In this report, we present the draft genome sequence of the S. bacillaris type strain CBS 9494, isolated from sweet botrytized wines, which will contribute to the study of this genetically heterogeneous wine yeast species.

## ANNOUNCEMENT

Starmerella bacillaris (syn., Candida zemplinina) (1) is a non-Saccharomyces yeast ubiquitously present in oenological environments (2–7) and occasionally recognized in soil, fruit insects, and some rotten fruits (5, 8, 9). The species, first recognized in Napa Valley (USA) in *Botrytis*-affected wine fermentations (10), was then identified through a detailed morphological, physiological, and molecular characterization of the S. bacillaris type strain 10-372 (=CBS 9494^T^=NCAIM Y016667^T^), isolated from white wine in Zemplin, Hungary (11). The potential use of S. bacillaris in winemaking has been studied in mixed fermentations with Saccharomyces cerevisiae, where its fructophilic character and ability to produce wines with reduced ethanol levels represent potential advantages (12–19). Sensory evaluation revealed that pure starters of S. cerevisiae were preferred to mixed S. cerevisiae/S. bacillaris fermentations (20). Phylogenetic analyses of S. bacillaris strains isolated from winemaking environments showed that neither clonal-like behavior nor specific genetic signatures were associated with strain populations at the different analyzed vineyards and wineries (21). These studies suggest that S. bacillaris is not under selective pressure in winemaking environments since its genetic diversity is shaped by geographical localization (21). The availability of the genome sequence of the S. bacillaris type strain CBS 9494 reported here, as well as additional specific molecular tools for population analyses (5, 15, 21–23), will contribute to the knowledge of the genetic and geographic diversity of this species as well as its life cycle (21).

In this study, total DNA from S. bacillaris type strain CBS 9494 was prepared using a genomic DNA buffer set and Genomic-tip 100/G kits (Qiagen). Genomic DNA (1 μg) was enzymatically fragmented (10 min) and size selected (315 bp) using the Pippin Prep instrument (Sage Science); a genomic library was generated with the Ion Xpress Plus fragment library kit (ThermoFisher Scientific). The genome sequence (122-fold genome coverage), obtained on the Ion Torrent PGM platform (Life Technologies), was trimmed based on a Phred-type quality threshold score of Q20 (QPhred = 20) and a length threshold of 50 bp using CLC Genomics Workbench version 7.0.3 (CLC bio). A total of 5,698,579 reads (mean size of 200 bp) were used for *de novo* assembly with Newbler version 2.7 (454 Life Sciences), which resulted in 200 contigs longer than 200 bp (mean, 108,648 bp; maximum, 649,352 bp; *N*_50_, 175,910 bp). The genome of S. bacillaris type strain 9494 was 9.3 Mb with a G+C content of 39.4%, similar to the recently available genome data for S. bacillaris strains FRI751 (24) and PAS13 (25). The S. bacillaris type strain CBS 9494 genome sequence reported here will constitute a reference sequence for studying evolutionary and phylogenetic aspects, as well as the geographic biodiversity, of this genetically heterogeneous species (21).

### Data availability.

This whole-genome shotgun project has been deposited at DDBJ/ENA/GenBank under the accession number QLKO00000000. The version described in this paper is the first version, QLKO01000000.
