# Correction for Rosa et al., “Draft Genome Sequence of the *Candida zemplinina* (syn., *Starmerella bacillaris*) Type Strain CBS 9494”

**DOI:** 10.1128/MRA.01116-18

**Published:** 2018-09-13

**Authors:** Alberto Luis Rosa, Cécile Miot-Sertier, Yec’han Laizet, Franck Salin, Matthias Sipiczki, Marina Bely, Isabelle Masneuf-Pomarede, Warren Albertin

**Affiliations:** a Laboratorio de Genética y Biología Molecular, IRNASUS-CONICET, Facultad de Ciencias Químicas, Universidad Católica de Córdoba, Córdoba, Argentina; b ISVV, Oenology Research Unit EA 4577, USC 1366 INRA, Bordeaux INP, University of Bordeaux, Bordeaux, Villenave d’Ornon, France; c UMR Biodiversity of Genes and Ecosystems, Genomics Platform, INRA, Cestas, France; d Department of Genetics and Applied Microbiology, University of Debrecen, Debrecen, Hungary; e Bordeaux Sciences Agro, Gradignan, France; f ENSCBP, Bordeaux INP, Pessac, France

## AUTHOR CORRECTION

Volume 7, no. 3, e00872-18, 2018, https://doi.org/10.1128/MRA.00872-18. Page 1: The article title should read as given above.

Page 1, line 1: “*Starmerella bacillaris* (syn., *Candida zemplinina*)” should read “*Candida zemplinina* (syn., *Starmerella bacillaris*).”

Pages 1 and 2: “*S. bacillaris*” should read “*C. zemplinina*” throughout.

